# Doubts about the diagnosis and treatment of syphilis in pregnancy among primary care professionals in a telehealth service

**DOI:** 10.1371/journal.pone.0306192

**Published:** 2024-06-28

**Authors:** Renata Rosa de Carvalho, Fabiana Carvalho, Elise Botteselle de Oliveira, Rodolfo Souza da Silva, Dimitris Varvaki Rados, Rita Mattiello, Marcelo Rodrigues Gonçalves, Roberto Nunes Umpierre, Camila Giugliani

**Affiliations:** 1 Núcleo de Telessaúde do Rio Grande do Sul (TelessaúdeRS-UFRGS), Federal University of Rio Grande do Sul, Porto Alegre, Brazil; 2 Post Graduate Studies Program in Epidemiology, School of Medicine, Federal University of Rio Grande do Sul (UFRGS), Porto Alegre, RS, Brazil; 3 Social Medicine Department, Federal University of Rio Grande do Sul, Porto Alegre, Brazil; Hospital Femina, BRAZIL

## Abstract

This cross-sectional study aims to describe doubts regarding the diagnosis and treatment of syphilis in pregnancy among primary care professionals in a telehealth service. All teleconsultations (TCs) offered through TelessaúdeRS-UFRGS to primary health care (PHC) services in the state of Rio Grande do Sul between 2018 and 2021 involving syphilis in pregnancy were included. A total of 356 (TCs) were analyzed. The main doubts about syphilis during pregnancy raised by primary care professionals were related to the need for retreatment (35%), diagnostic definition (23%) and initial treatment (16%). In addition, 95% of TCs were suitable for diagnosing and treating syphilis based on the 2020 Brazilian Ministry of Health guideline. This study suggests that TCs can identify failures in the diagnosis and treatment of public health problems and support decision making in PHC involving syphilis in pregnancy.

## Introduction

Syphilis in pregnancy and congenital syphilis are major public health problems worldwide [1–3]. Treatment of syphilis is effective, and several international and local guidelines exist for managing this condition. However, every year, there are about 6 million new cases of syphilis among people aged 15 to 49 worldwide. In addition, nearly 300,000 fetal and neonatal deaths are attributable to syphilis, and 215,000 children are at risk of premature death annually due to congenital syphilis [1, 2]. In Brazil, the scenario is also concerning in 2022, the total number of cases of syphilis in pregnancy was 83,034 [4].

There are several possible explanations for this situation, including misdiagnosis of acquired syphilis, which could increase the number of untreated transmitters in the population, failure to initiate or complete treatment, and non-treatment of sexual partners [1, 3, 4]. Social vulnerability is among the main risk factors for non-adherence to individual therapy [1, 3].

The Brazilian Ministry of Health develops and consistently updates guidelines to aid in the diagnosis and treatment of syphilis. Nonetheless, the lack of adherence to these guidelines in primary care settings also contributes to the prevailing statistics [1, 5]. Further research is essential on this topic, particularly in settings where primary care professionals can call upon the support of telehealth services.

Telehealth technologies and strategies have improved infectious disease care, including screening, medication adherence, the number of patients lost to follow-up, attendance at scheduled medical appointments, and costs, particularly for patients with HIV and viral hepatitis [6, 7]. In addition, such innovations have reduced gaps in access to care for marginalized, geographically isolated populations or those lacking technological infrastructure [8, 9]. One of the main reported benefits of telehealth has been increased access to specialist care, particularly for isolated populations, including residents of long-term care facilities [10]. Another advantage of the telehealth service is the identification of failures in the diagnosis and treatment of public health problems. This identification can offer insights into areas where additional education of health care teams is required.

### Aims of the study

The present study aims to describe doubts regarding the diagnosis and treatment of syphilis in pregnancy among primary care professionals in a telehealth service. A secondary study objective was to describe the diagnostic and treatment adequacy of teleconsultations (TCs) to the Brazilian Ministry of Health guideline for syphilis and the percentage of referrals to a specialist in patients with syphilis in pregnancy who were treated in primary health care (PHC) and supported by a telehealth service.

## Methods

### Study design

This cross-sectional study included an analysis of retrospective records from the telehealth department to investigate the nature of clinical inquiries about syphilis in pregnancy. This study followed the STrengthening the Reporting of OBservational studies in Epidemiology (STROBE) method [11].

### Setting

TelessaúdeRS-UFRGS is affiliated with the Federal University of Rio Grande do Sul (UFRGS). The telehealth program has already been detailed elsewhere [10, 12, 13]. Briefly, the TC modality allows providers to discuss in real time specific patient- or practice-related questions concerning all primary care issues, including infectious diseases. In a synchronous interaction, a TelessaúdeRS-UFRGS teleconsultant receives a query from a primary health care (PHC) provider, searches for the best evidence to answer the question, and, when necessary, requests support from another teleconsultant. To find the best evidence, the telehealth advisors are trained to follow a sequence of searches for any query from general practitioners, starting with national guidelines [5] and TelessaúdeRS guidelines (e.g. syphilis ebook), followed by textbooks and electronic portals (Dynamed, UpToDate).

Once an answer has been prepared, the teleconsultant verbally provides the inquirer with a suggestion for management, while also making a written record of the information on a text platform [12, 13]. Each case attended in TCs is recorded, and the primary care professional can carry out further consultations on the same case if necessary.

The syphilis questions were mostly answered by generalists (family physicians and internal medicine specialists). TelessaúdeRS-UFRGS consultants don’t receive specific training for syphilis, but they are instructed to follow the most current TelessaúdeRS and Ministry of Health guidelines for diagnosing and treating the disease. Furthermore, since syphilis during pregnancy is a primary care sensitive problem, the telehealth service coordinators routinely discuss the TCs provided to maintain quality of care. The hotline coordinators are experienced family physicians, and they perform periodic feedback interviews with the telehealth advisors. In these interviews, the advisors are informed about the quality of their answers and if they follow the recommended materials and sequence of literature searches. Also, as mentioned above, there is an opportunistic review of inaccurate answers, sometimes during the call. When incorrect or inaccurate answers are detected, additional calls are made to complete or update the information provided to the general practitioners.

### Participants

This study only included TelessaúdeRS-UFRGS TCs related to syphilis in pregnancy offered to primary care providers between 2018 and 2021. Data were accessed for research purposes in January 2022 and analyzed between February and April 2022. Syphilis cases could be primary, secondary, tertiary, recent latent, late latent, unknown duration, and congenital syphilis.

The selection of TCs was carried out in three stages, using convenience sampling. All TCs in Rio Grande do Sul (RS) were selected during the first stage. The next phase included only consultations about syphilis. Finally, during the third stage, only those about syphilis in pregnancy were included.

### Variables

The variables included in this study were collected from the Rio Grande do Sul TelessaúdeRS-UFRGS database and were as follows: age of the pregnant patient on the date of the TCs (years); stage (trimester) of pregnancy; the patient’s place of residence; history of syphilis before pregnancy (yes or no); time of TCs (month/year); method used to diagnose syphilis (rapid treponemal test, nontreponemal test, other treponemal tests, both nontreponemal and treponemal tests, or no laboratory diagnosis); reason why previous treatment was considered inappropriate (wrong dose, wrong interval between doses, wrong drug, no record of treatment, or no indication for treatment) (S1 File); HIV testing (not performed or not reported, positive/reactive, or negative/nonreactive); partner testing for syphilis (not performed or not reported, positive/reactive, or negative/nonreactive); and partner treatment for syphilis (yes, no, or not reported). To characterize the profile of providers who requested TCs, we collected the following data from the National Registry of Health Facilities: occupational category, occupation (according to the Brazilian codes of professions), municipality, and type of health facility.

### Main outcomes

The primary outcome of the study was doubts regarding the diagnosis and treatment of syphilis in pregnancy among primary care professionals in the TelessaúdeRS-UFRGS TCs.

We also describe the adequacy of TCs diagnosis and treatment to the Brazilian Ministry of Health guideline for syphilis (S1 File) and their characteristics. This guideline, entitled "The Brazilian Ministry of Health Clinical Protocol and Practice Guidelines for Prevention of Mother-to-Child Transmission of HIV, Syphilis, and Viral Hepatitis" [5], establishes diagnostic criteria for sexually transmitted infections (STIs) as well as recommended treatment, prevention, and follow-up strategies for these diseases. It is periodically reviewed based on the latest evidence and considers efficacy, safety, and cost-effectiveness criteria. All information about the diagnostic and treatment recommendations used in the guideline is described in S1 File. The evaluation of the adequacy of TCs diagnosis and treatment to the Brazilian Ministry of Health guideline for syphilis was carried out by a family physician who is a certified auditor of TCs services at TelessaúdeRS-UFRGS.

### Statistical methods

PASW Statistics for Windows, version 18.0. (Chicago: SPSS Inc.) was used for the data analysis. The data were expressed as mean (SD) for the continuous variables and absolute and relative frequencies for the categorical variables. All TCs about syphilis in pregnancy performed during the analysis period were included.

### Ethical approval

The present study followed the standards for research involving human subjects, as outlined in Brazilian National Health Council Resolution 466/2012, and complied with the General Data Protection Law. The study was approved by the Research Ethics Committee of Hospital de Clínicas de Porto Alegre (CAAE 52032721.6.0000.5327 and Project 2021–0444) and was conducted according to the Declaration of Helsinki. The Ethics Committee waived the need for informed consent, since our objective was to describe the synchronous provider-to-provider TCs service. Additionally, all data in the database were fully anonymized, which ensured that participants’ identities could not be traced during analysis.

## Results

**Fig 1:**
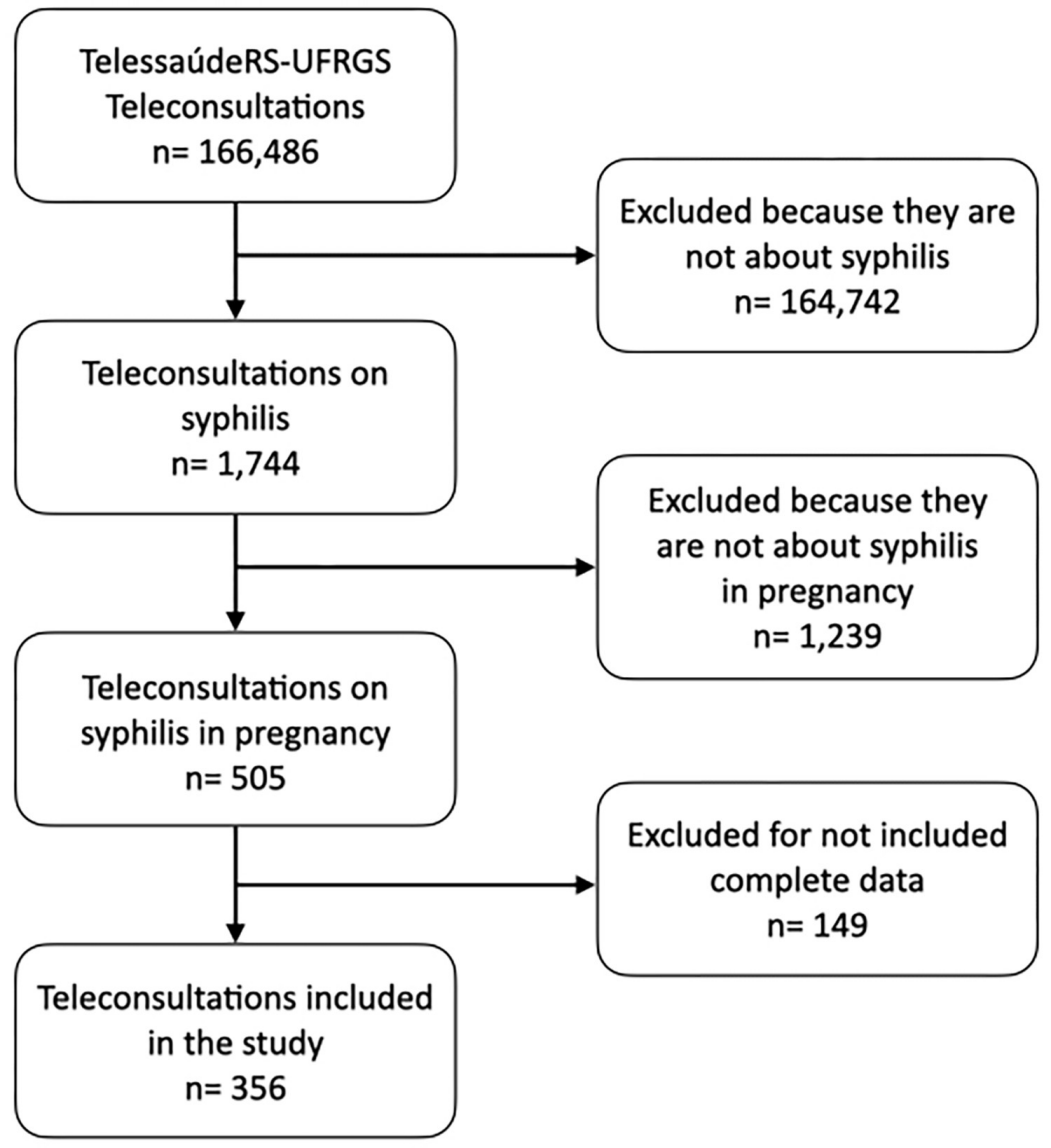
Flowchart of TCs carried out via TelessaúdeRS-UFRGS.

Of the 166,486 TCs carried out via TelessaúdeRS-UFRGS during the period studied, 505 were about syphilis during pregnancy and 356 were included in the study (Fig 1). Two hundred and seven had one TC to discuss the case and 149 had two or more. The year with the highest number of TCs analyzed was 2020, accounting for 46% of the TCs included in the study, representing an increase of 58% in TCs compared to the previous year. Of the 497 municipalities in the state of Rio Grande do Sul, 85 had at least one TC about syphilis in pregnancy performed during the analysis period. The Metropolitan health macro-region, which encompasses the area surrounding the state capital city, accounted for 257 TCs (72%).

The mean age of the patients was 25 (SD±5.9) years. Most of the patients did not have a history of syphilis before pregnancy 125 (65%) and were in the second 130 (36.5%) or third 143 (40%) trimester of pregnancy. Table 1 shows the characteristics of the management of cases of syphilis in pregnancy in Rio Grande do Sul in the context of primary care that were discussed with TelessaúdeRS. The most commonly used tests for diagnosing syphilis were the treponemal (33%) and nontreponemal tests (41%). Regarding treatment, 60% received syphilis treatment before the TC, and the treatment was appropriate according to clinical classification in 80% of cases. The main treatment failures were dose related.

**Table 1:** Characteristics of the management of cases of syphilis in pregnancy in Rio Grande do Sul primary care discussed with TelessaúdeRS.

Characteristics	TCs on syphilis (n = 356)
	Mean (SD)
Diagnosis of syphilis during current pregnancy	
Rapid treponemal test	118 (33.1%)
Nontreponemal test	147 (41.3%)
Other treponemal test (not RT)	3 (0.8%)
Both nontreponemal and treponemal tests	69 (19.4%)
No laboratory diagnosis	19 (5.3%)
Reason to consider treatment inappropriate (n = 43)	
Incorrect dose	31 (72.1%)
Incorrect interval between doses	1 (2.3%)
Inappropriate drug	1 (2.3%)
No record of treatment	4 (9.3%)
No indication for treatment	6 (14%)
HIV testing	
Not performed or not reported	279 (78.4%)
Positive/Reactive	3 (0.8%)
Negative/Nonreactive	74 (20.8%)
Partner testing for syphilis	
Not performed or not reported	242 (68.0%)
Positive/Reactive	46 (12.9%)
Negative/Nonreactive	68 (19.1%)
Partner treated for syphilis.	
Yes	104 (29.2%)
No/Not reported	252 (70.8%)

Values are described as mean and standard deviation (SD) or frequency (%). TCs: teleconsultations

* Treatment adequacy according to Brazilian Syphilis Ministry of Health guidelines.

Despite the recommendation of HIV screening for people diagnosed with sexually transmitted infections, in 78% of the cases it was not performed, or information on HIV testing was not given or not recorded. The same was true for information about syphilis testing and treatment for partners.

Table 2 shows the description of TCs on syphilis in pregnancy between 2018 and 2021. The main topic of requesting provider’s question was related to retreatment (35%). In 35% of TCs, the teleconsultant concluded that the pregnant patient with syphilis was receiving appropriate treatment; in 33%, the consultant’s impression was of late latent syphilis, latent syphilis of unknown duration, or tertiary syphilis; and in 10%, the patient met criteria for reinfection or treatment failure. In TCs that resulted in an indication for treatment or retreatment (45%), the most commonly recommended therapeutic regimen was benzathine benzylpenicillin in a single 2,400,000 IU dose (60%).

**Table 2:** Description of teleconsultations on syphilis in pregnancy between 2018 and 2021.

Description	TCs on syphilis in pregnancy (n = 356)
Topic of requesting provider’s question	
Retreatment	124 (34.8%)
Diagnosis	80 (22.5%)
Initial treatment	57 (16%)
Follow-up after diagnosis and treatment	42 (11.8%)
Partner treatment	30 (8.4%)
Need for referral to specialized care	13 (3.7%)
Administration of benzathine benzylpenicillin	8 (2.2%)
Notification/Reporting	1 (0.3%)
Appropriate treatment	1 (0.3%)
Impression of teleconsultant	
True syphilis in pregnancy, appropriately treated	125 (35.1%)
Late latent syphilis/latent syphilis of unknown duration/tertiary syphilis	117 (32.9%)
Reinfection or treatment failure	34 (9.6%)
Serofast reaction	28 (7.9%)
Exposure to a partner diagnosed with syphilis	18 (5.1%)
Primary/secondary/recent latent syphilis	16 (4.5%)
No syphilis/additional diagnostics required	15 (4.2%)
False positive	3 (0.8%)
Recommendation of teleconsultant	
No treatment recommended/follow-up only	193 (54.2%)
Treatment recommended	110 (30.3%)
Retreatment recommended	53 (14.9%)
Recommended course of treatment	
Benzathine benzylpenicillin, 2,400,000 IU	215 (60.4%)
Benzathine benzylpenicillin, 7,200,000 IU	141 (39.6%)
Doxycycline (for partner)	1 (0.3%)
TCs with follow-up for the same patient	149 (41.9%)
TCs was completed only after a discussion with senior teleconsultant	142 (39.9%)
TCs followed the Ministry of Health guideline	
Yes	338 (94.9%)
Level of care recommended by teleconsultant	351 (98.6%)

Values are described as frequency (%).

In 95% of TCs, the information provided by the teleconsultants was suitable for diagnosing and treating syphilis according to the Brazilian Ministry of Health guideline. The main ways the TCs did not follow the Ministry of Health guideline (n = 18) were by not recommending treatment in cases that met treatment or retreatment criteria, recommending treatment or retreatment in the absence of established standards, or providing the incorrect medication dose. The decision in most TCs (98.6%) was to maintain follow-up in PHC. The main reason for referral to specialized care was the need to investigate neurosyphilis. One pregnant patient was referred to the emergency department for penicillin desensitization due to a history of severe allergy.

## Discussion

The present study identified that there are significant flaws in the diagnosis and treatment of syphilis in pregnancy, despite the national guideline and resources available for this problem in primary health care. Recent evidence shows an increase in syphilis cases in Brazil in the last few years and highlights the need to plan and develop effective multidisciplinary prevention actions and improved prenatal care [14].

This increase in the disease has been associated with several factors, such as population disinformation and sociodemographic disparities, race, maternal age, benzatine penicillin shortages, and absence prenatal care prenatal care [15–17]. In addition to implementing health care actions integrated with surveillance and control, ensuring access to timely PHC diagnosis, treatment, and monitoring has been the greatest challenge to achieving control of the disease [15–18].

The present study also suggests the need for professional training in PHC for diagnosing, treating, and providing counseling for syphilis in pregnancy, as well as the importance of programs such as telehealth as a support and educational tool for primary care [16–18]. In the case of syphilis in pregnancy, proper diagnosis and treatment are associated with taking care of the health of the mother and her partner and can prevent congenital syphilis [15]. This is imperative because there is a high heterogeneity in Brazilian PHC, both in the training of human resources and the quality of care provided [14].

Most of the TCs provided by the TelessaúdeRS-UFRGS program followed the guideline for diagnosing and treating gestational syphilis [5]. In addition, less than two percent of TCs resulted in a referral to specialized care. These results suggest the high potential of TCs to support point-of-care decision making based on guidelines, particularly those offered nationally. Following the Ministry of Health’s syphilis guidelines, TelessaúdeRS-UFRGS has given PHC providers more assertive access to up-to-date information on syphilis in pregnancy, ensuring guidance according to the latest evidence-based recommendations for nearly all cases of syphilis during pregnancy.

Evaluation of a health program or intervention must include structure, process, and outcome indicators [16, 18]. In this logic, the outcome variables chosen for this study–adequacy of guidance and TCs decision–can be characterized as process and outcome indicators, respectively [19].

In 2020, there was an 80% increase in demand for TCs at TelessaúdeRS-UFRGS, and during this period there was an almost 60% increase in queries about syphilis compared to 2019 [12]. One of the reasons for this increase may have been the COVID-19 pandemic, since–especially in those months with the highest incidence of syphilis cases–the supply of elective or non-emergency care was disrupted, despite the Ministry of Health’s guidance to maintain a regular provision of antenatal care. This may have increased the use of telehealth to optimize time and ensure appropriate case management in the context of fragmented longitudinal follow-up [19, 20].

Despite several studies highlighting the importance of telehealth as a means of support for care providers, including in remote areas, regions farther away from large urban centers still show a low frequency of use of this technology, as verified in the present study, which showed that most requesting providers worked in the largest metropolitan region of the state [21].

Only recently has more significant progress been made in overcoming the legal and political restrictions that govern the development of information technology in Brazil, due to the needs imposed by the COVID-19 pandemic [12, 13, 22–24]. Despite its increasing use in the country, telehealth still lacks strong, permanent guidelines on funding models and regulatory frameworks in Brazil [19].

This study is not without limitations. First, it only included data from primary care professionals who requested the TCs service and from one Brazilian state, using convenience sampling; generalization of the results should be done with caution. However, Porto Alegre, the capital of the state of Rio Grande do Sul, is one of the cities with the highest prevalence of syphilis. In 2021, more than 167,000 new cases of acquired syphilis and 74,000 cases among pregnant women were registered in Brazil, with another 27,000 cases of congenital syphilis being diagnosed. In the same year, Porto Alegre reported 1,914 cases of acquired syphilis, 1,141 cases among pregnant women, and 610 cases of congenital syphilis. These numbers should be higher because of underreporting [5].

The study did not assess the follow-up of TCs where PHC providers were advised by teleconsultants. Future studies could include data from Brazil and monitor patients to evaluate the effects of teleconsultations on outcomes associated with syphilis in pregnancy. However, high adherence to guidelines increases the likelihood of better case resolution. Another limitation to be considered is that part of the data collection was carried out during the period of the COVID-19 pandemic, which affected primary care services. However, there is consistent evidence describing an increase in syphilis cases, particularly in Brazil, in the last ten years [15]. Finally, the study did not include information from TCs with missing data.

The present study offers insights into areas requiring further education for health care teams–such as diagnosis, retesting, and understanding treatment failures. It also highlights the complex nature of gestational syphilis and solutions, such as telehealth consultation, to aid healthcare providers in handling specific patient cases.

## Supporting information

S1 File Main recommendations of the Brazilian Health Ministry for syphilis test interpretation, diagnosis, and management in pregnant women. (DOCX)
