# Supplementary material for: Doubts about the diagnosis and treatment of syphilis in pregnancy among primary care professionals in a telehealth service
Source: PLoS One. 2024 Jun 28;19(6):e0306192. doi: 10.1371/journal.pone.0306192 (PMC11213312; doi:10.1371/journal.pone.0306192)
Supplement: S1 File — (DOCX) [file pone.0306192.s001.docx]

**S1 File.** Main recommendations of the Brazilian Health Ministry for syphilis test interpretation, diagnosis, and management for pregnant women.

| Test interpretation and clinical conduct | | | | |  |
| --- | --- | --- | --- | --- | --- |
| Initial test |  | Additional test | Interpretation | Management |  |
| **Treponemal test Reagent** | **+** | **Non-treponemal test Reagent** | Diagnosis of syphilis. Classification is to be defined according to the time of infection and treatment history.   If there is a history of adequate treatment and adequate immune response, it may represent a lack of seroreversion. | If syphilis diagnosis:  - Treat and monitor response  - monthly follow-up with non-treponemal  - Public health authority notification  If lack of seroreversion: patient education. |  |
| **Treponemal test Reagent** | **+** | **Non-treponemal test Non-reagent** | Perform an additional treponemal test with a different methodology from the initial test. If **negative**, consider it a false-positive, and the diagnosis is discarded.  If **positive**, evaluate if there is documentation of previous syphilis treatment. If not documented, untreated, or unknown, manage as recent syphilis. | If syphilis diagnosis:  - Treat and monitor response  - monthly follow-up with non-treponemal  - Public health authority notification  If lack of seroreversion or syphilis discarded: patient education. |  |
| **Non-treponemal test Reagent** | **+** | **Treponemal test Reagent** | Diagnosis of syphilis. Classification is to be defined according to the time of infection and treatment history.   If there is a history of adequate treatment and adequate immune response, it may represent a lack of seroreversion. | If syphilis diagnosis:  - Treat and monitor response  - monthly follow-up with non-treponemal  - Public health authority notification  If lack of seroreversion: patient education. |  |
| **Non-treponemal test Reagent** | **+** | **Treponemal test Non-reagent** | There is a probable false-positive in the non-treponemal test, especially if the titer is 1:4 or less. Perform an additional treponemal test with a different methodology from the initial test. If **negative**, consider it as false-positive, and the diagnosis is discarded.  If **positive**, evaluate if there is documentation of previous syphilis treatment. If not documented, untreated, or unknown, manage as recent syphilis. | If syphilis diagnosis:  - Treat and monitor response  - monthly follow-up with non-treponemal  - Public health authority notification  If lack of seroreversion or syphilis discarded: patient education. |  |
| **Non-treponemal test or Treponemal test  Non-reagent** | **+** | Do not perform a complementary test if the first test is **Non-reagent** and there is no clinical suspicion of primary syphilis. | Absence of infection. Consider the possibility of false-negative results in patients with clinical manifestations of early syphilis. | In case of clinical manifestations and or epidemiological suspicion, request a test within 30 days. Do not delay treatment if the diagnosis is highly suspected or there is a risk of losing follow-up. |  |
| Treatment | | | | |  |
| Regimens for pregnant women | | | | |  |
| Recent syphilis (with less than one year of evolution) *: primary, secondary, and recent latent syphilis | | | Penicillin G benzathine is 2.4 million units of intramuscular injection-dose single (1.2 million units in each buttock). | |  |
| Late syphilis (with more than one year of evolution) *: late latent or latent syphilis with duration ignored and tertiary syphilis | | | Penicillin G benzathine is 2.4 million units of intramuscular injection (1.2 million units in each buttock) weekly for three weeks. Total dose: 7.2 million units intramuscular. | |  |
| Neurosyphilis | | | Crystalline penicillin 18-24 million units/day intravenous. Use doses of 3-4 million units every 4 hours or continuous infusion for 14 days. | |  |
| Additional recommendations | | | | |  |
| Sexual partners recommendations | | | Sexual partners may be infected even with non-reactive laboratory tests. Therefore, they should be treated with one dose of benzathine penicillin (2.4 million units intramuscular). If the laboratory test for syphilis is reactive, follow the recommendations for acquired syphilis in adults using preferably benzathine penicillin. | |  |
| The following criteria indicate adequate syphilis treatment in pregnancy | | | Treatment with benzathine penicillin following the recommendations for the clinical stage. Treatment starts 30 days before the delivery. The treatment is maintaining the administration within the recommended schedule. Evaluation of the reinfection risk. | |  |
| **During the study period, the classification of syphilis was updated according to Ministry of Health protocols. Between 2017 and 2019, 2 years were used to define recent or late stages.* | | | | |  |
|  |  |  |  |  |  |
